# Synaptic Tuning of Brain Rhythms: From Chemical Signalling to Cortical Oscillations

**DOI:** 10.1002/hbm.70463

**Published:** 2026-02-02

**Authors:** Maxime O. Baud, Dimitri Van De Ville

**Affiliations:** ^1^ Sleep‐Wake‐Epilepsy Center, NeuroTec, Center for Experimental Neurology, Department of Neurology Inselspital Bern, University Hospital, University of Bern Bern Switzerland; ^2^ Neuro‐X Institute Ecole polytechnique fédérale de Lausanne (EPFL) Geneva Switzerland; ^3^ Department of Radiology and Medical Informatics University of Geneva Geneva Switzerland

## Abstract

Stoof et al. investigate why distinct brain regions exhibit characteristic oscillatory frequencies, such as occipital alpha and frontal beta rhythms. Their work elegantly links openly available intracranial EEG spectra from 106 epilepsy patients to synaptic receptor densities from available autoradiography maps in three healthy donors. In the framework of dynamic causal modelling, they show that regional oscillations emerge from balanced combinations of excitatory (AMPAR, NMDAR) and inhibitory receptors (GABAR A or B), while neuromodulatory receptors exert subtler influences.

## Graphical abstract

Synaptic tuning of brain rhythms. A network of neurons communicating via different synaptic receptors may give rise to brain oscillations at different frequencies in different brain regions. Left: schematic network of neurons communicating via different synaptic receptors. Right: brain oscillations at different frequencies in different brain regions coloured by the corresponding red, green blue mix of synaptic receptors. Images generated with biorender.com.

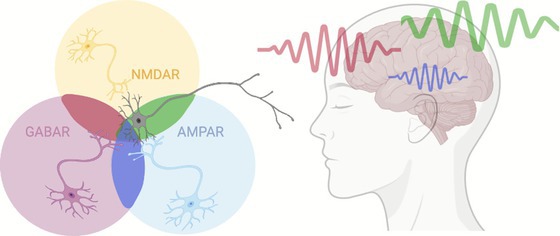

Why does the alpha rhythm predominate in the occipital lobe and beta oscillations characterize the frontal cortex? Could regional variations in the excitatory‐inhibitory ratio and its tuning by neuromodulators account for these differences?

These are the key questions that Stoof et al. elegantly sought to address in an original study entitled “Topographic variation in human neurotransmitter receptor densities explains differences in intracranial EEG spectra” in this issue (Stoof et al. 2025).

Their work essentially explores how synaptic receptor densities may shape regional brain oscillations, leveraging several assets. First, it relies on existing autoradiography maps of 15 ionotropic or metabotropic synaptic receptors from three human donors aged 72–77 (Zilles and Palomero‐Gallagher 2017), here interpolated and integrated into a shared brain template alongside available power spectra of brain oscillations from 106 patients with epilepsy aged 33 ± 11 years (Frauscher et al. 2018). The latter were derived from previously published intracranial EEG data, recorded directly from non‐epileptic brain regions among 106 patients who underwent a presurgical work‐up of their epilepsy with penetrating electrodes. Arguably, such iEEG recordings are the best currently available, as they exquisitely capture brain oscillations with millimeter and millisecond precision. Second, in their search for correlations between recorded oscillations and receptor densities, the authors used the dynamic causal modelling (DCM) framework. Therein, coupled neural mass models include excitatory and inhibitory populations, and account for neuromodulatory effects to produce oscillatory behaviour. Specifically, canonical microcircuit models were fitted to the iEEG traces, and a Bayesian approach was used to incorporate the influence of multiple receptors and improve information gain. As a validation approach, a null model in which synaptic receptor maps were shuffled was considered. In their original work, the authors convincingly use mesoscale models (coupled neural masses) to link micro‐scale synaptic properties (receptor densities) to macro‐scale functional properties, namely brain oscillations at specific frequencies.

The main finding of Stoof et al. is that no single neurotransmitter receptor explains any specific oscillatory frequency in isolation. Instead, regional oscillatory spectra arise from a balanced combination of synaptic receptors, summarised as four principal components in a data‐driven decomposition of receptor density maps. The inclusion of densities of three primary synaptic receptors—AMPAR, NMDAR, and GABAR (A or B)—significantly improved the fit of canonical neural mass models to better render oscillatory spectra compared to null and baseline configurations, suggesting a dominant role of the excitatory/inhibitory balance in setting oscillation frequency. The addition of receptors mediating neuromodulatory control by acetylcholine, dopamine, serotonin, and noradrenaline only marginally enhanced the fits, suggesting that neuromodulation exerts subtler, likely context‐dependent effects on momentary brain oscillations.

As with any pioneering multimodal study, several limitations exist and present opportunities for future research. A primary caveat is that spatially sparse iEEG data and receptor densities were obtained from different cohorts, necessitating ample interpolation of both datasets into a common MNI template space. While such spatial normalisation is standard practice in MRI research, its emerging application to iEEG poses additional problems because recordings are very sparse and biased towards sampling of epileptic brain regions. Here, iEEG traces were recorded over only 60 s of wakefulness from 1770 electrodes crossing relatively healthy brain tissue on their way to putative seizure foci (Frauscher et al. 2018). The data did not include actual probing of the excitability in different brain regions, which is achievable with iEEG (Friedrichs‐Maeder et al. 2025). Receptor density data (Zilles and Palomero‐Gallagher 2017) was limited to post‐mortem in vitro layer‐specific receptor autoradiography from three healthy, but aged donors. Thus, this limited static map of synaptic receptors cannot reflect the variability in synaptic receptor density possibly found in a broader and/or younger population. To scale up the approach, future similar work could leverage existing PET (positron emission tomography) atlases of synaptic receptors collated among 1200 individuals (Hansen et al. 2022) or magnetic resonance spectroscopy imaging (MRSI) data that is emerging as a complementary modality to probe neurotransmitter content at the timescale of minutes (Kasten et al. 2016; Bogner et al. 2021).

Furthermore, the canonical microcircuit model used for DCM did not explicitly include neuromodulatory synapses, nor a connectome. In each brain region, iEEG sampled a few nodes (here, electrodes), and the recorded data were represented as fixed spectra, thereby capturing the stationary characteristics of regional oscillations in isolation, but not the temporally evolving nature of coupled oscillatory networks.

In sum, Stoof et al. provide a compelling step forward in our understanding of synaptic tuning of brain rhythms, which will undoubtedly stimulate refined efforts to integrate receptor mapping, iEEG, and network modelling into a unified framework of brain function and dysfunction. Ultimately, apprehending the spatiotemporal unfolding of brain oscillations will require moving beyond static maps of neuromodulators and integrating them into models that capture the full electrochemical and network complexity of the human brain.

## Funding

The authors have nothing to report.

## Conflicts of Interest

The authors declare no conflicts of interest.

## Data Availability

Data sharing not applicable to this article as no datasets were generated or analysed during the current study.
